# Predicting at-risk opioid use three months after ed visit for trauma: Results from the AURORA study

**DOI:** 10.1371/journal.pone.0273378

**Published:** 2022-09-23

**Authors:** Brittany E. Punches, Uwe Stolz, Caroline E. Freiermuth, Rachel M. Ancona, Samuel A. McLean, Stacey L. House, Francesca L. Beaudoin, Xinming An, Jennifer S. Stevens, Donglin Zeng, Thomas C. Neylan, Gari D. Clifford, Tanja Jovanovic, Sarah D. Linnstaedt, Laura T. Germine, Kenneth A. Bollen, Scott L. Rauch, John P. Haran, Alan B. Storrow, Christopher Lewandowski, Paul I. Musey, Phyllis L. Hendry, Sophia Sheikh, Christopher W. Jones, Michael C. Kurz, Nina T. Gentile, Meghan E. McGrath, Lauren A. Hudak, Jose L. Pascual, Mark J. Seamon, Erica Harris, Anna M. Chang, Claire Pearson, David A. Peak, Roland C. Merchant, Robert M. Domeier, Niels K. Rathlev, Brian J. O’Neil, Leon D. Sanchez, Steven E. Bruce, Robert H. Pietrzak, Jutta Joormann, Deanna M. Barch, Diego A. Pizzagalli, Jordan W. Smoller, Beatriz Luna, Steven E. Harte, James M. Elliott, Ronald C. Kessler, Kerry J. Ressler, Karestan C. Koenen, Michael S. Lyons

**Affiliations:** 1 College of Nursing, The Ohio State University, Columbus, OH, United States of America; 2 Department of Emergency Medicine College of Medicine, The Ohio State University, Columbus, OH, United States of America; 3 Department of Emergency Medicine, University of Cincinnati, Cincinnati, OH, United States of America; 4 Center for Addiction Research, University of Cincinnati College of Medicine, Cincinnati, OH, United States of America; 5 Department of Emergency Medicine, Washington University School of Medicine, St. Louis, MO, United States of America; 6 Department of Emergency Medicine, University of North Carolina at Chapel Hill, Chapel Hill, NC, United States of America; 7 Department of Anesthesiology, Institute for Trauma Recovery, University of North Carolina at Chapel Hill, Chapel Hill, NC, United States of America; 8 Department of Emergency Medicine & Department of Health Services, Policy, and Practice, The Alpert Medical School of Brown University, Rhode Island Hospital and The Miriam Hospital, Providence, RI, United States of America; 9 Department of Psychiatry and Behavioral Sciences, Emory University School of Medicine, Atlanta, GA, United States of America; 10 Department of Biostatistics, Gillings School of Global Public Health, University of North Carolina, Chapel Hill, NC, United States of America; 11 Departments of Psychiatry and Neurology, University of California San Francisco, San Francisco, CA, United States of America; 12 Department of Biomedical Informatics, Emory University School of Medicine, Atlanta, GA, United States of America; 13 Department of Biomedical Engineering, Georgia Institute of Technology and Emory University, Atlanta, GA, United States of America; 14 Department of Psychiatry and Behavioral Neurosciences, Wayne State University, Detroit, MA, United States of America; 15 Institute for Technology in Psychiatry, McLean Hospital, Belmont, MA, United States of America; 16 The Many Brains Project, Belmont, MA, United States of America; 17 Department of Psychiatry, Harvard Medical School, Boston, MA, United States of America; 18 Department of Psychology and Neuroscience & Department of Sociology, University of North Carolina at Chapel Hill, Chapel Hill, NC, United States of America; 19 Department of Psychiatry, McLean Hospital, Belmont, MA, United States of America; 20 Department of Emergency Medicine, University of Massachusetts Medical School, Worcester, MA, United States of America; 21 Department of Emergency Medicine, Vanderbilt University Medical Center, Nashville, TN, United States of America; 22 Department of Emergency Medicine, Henry Ford Health System, Detroit, MI, United States of America; 23 Department of Emergency Medicine, Indiana University School of Medicine, Indianapolis, IN, United States of America; 24 Department of Emergency Medicine, University of Florida College of Medicine -Jacksonville, Jacksonville, FL, United States of America; 25 Department of Emergency Medicine, Cooper Medical School of Rowan University, Camden, NJ, United States of America; 26 Department of Emergency Medicine, University of Alabama School of Medicine, Birmingham, AL, United States of America; 27 Department of Surgery, Division of Acute Care Surgery, University of Alabama School of Medicine, Birmingham, AL, United States of America; 28 Center for Injury Science, University of Alabama at Birmingham, Birmingham, AL, United States of America; 29 Department of Emergency Medicine, Lewis Katz School of Medicine, Temple University, Philadelphia, PA, United States of America; 30 Department of Emergency Medicine, Boston Medical Center, Boston, MA, United States of America; 31 Department of Emergency Medicine, Emory University School of Medicine, Atlanta, GA, United States of America; 32 Department of Surgery, Department of Neurosurgery, University of Pennsylvania, Pennsylvania, PA, United States of America; 33 Perelman School of Medicine, University of Pennsylvania, Pennsylvania, PA, United States of America; 34 Department of Surgery, Division of Traumatology, Surgical Critical Care and Emergency Surgery, University of Pennsylvania, Pennsylvania, PA, United States of America; 35 Department of Emergency Medicine, Einstein Healthcare Network, Pennsylvania, PA, United States of America; 36 Department of Emergency Medicine, Sidney Kimmel Medical College, Thomas Jefferson University, Pennsylvania, PA, United States of America; 37 Department of Emergency Medicine, Jefferson University Hospitals, Pennsylvania, PA, United States of America; 38 Department of Emergency Medicine, Wayne State University, Detroit, MA, United States of America; 39 Department of Emergency Medicine, Massachusetts General Hospital, Boston, MA, United States of America; 40 Department of Emergency Medicine, Brigham and Women’s Hospital, Boston, MA, United States of America; 41 Department of Emergency Medicine, Saint Joseph Mercy Hospital, Ypsilanti, MI, United States of America; 42 Department of Emergency Medicine, University of Massachusetts Medical School-Baystate, Springfield, MA, United States of America; 43 Department of Emergency Medicine, Beth Israel Deaconess Medical Center, Boston, MA, United States of America; 44 Department of Emergency Medicine, Harvard Medical School, Boston, MA, United States of America; 45 Department of Psychological Sciences, University of Missouri—St. Louis, St. Louis, MO, United States of America; 46 National Center for PTSD, Clinical Neurosciences Division, VA Connecticut Healthcare System, West Haven, CT, United States of America; 47 Department of Psychiatry, Yale School of Medicine, New Haven, CT, United States of America; 48 Department of Psychology, Yale School of Medicine, New Haven, CT, United States of America; 49 Department of Psychological & Brain Sciences, Washington University in St. Louis, MO, United States of America; 50 Division of Depression and Anxiety, McLean Hospital, Belmont, MA, United States of America; 51 Department of Psychiatry, Psychiatric and Neurodevelopmental Genetics Unit, Massachusetts General Hospital, Boston, MA, United States of America; 52 Stanley Center for Psychiatric Research, Broad Institute, Cambridge, MA, United States of America; 53 Department of Psychiatry, University of Pittsburgh, Pittsburgh, PA, United States of America; 54 Department of Anesthesiology, University of Michigan Medical School, Ann Arbor, MI, United States of America; 55 Department of Internal Medicine-Rheumatology, University of Michigan Medical School, Ann Arbor, MI, United States of America; 56 Kolling Institute, University of Sydney, St Leonards, New South Wales, Australia; 57 Faculty of Medicine and Health, University of Sydney, Northern Sydney Local Health District, New South Wales, Australia; 58 Physical Therapy & Human Movement Sciences, Feinberg School of Medicine, Northwestern University, Chicago, IL, United States of America; 59 Department of Health Care Policy, Harvard Medical School, Boston, MA, United States of America; 60 Department of Epidemiology, Harvard T.H. Chan School of Public Health, Harvard University, Boston, MA, United States of America; University of South Australia, AUSTRALIA

## Abstract

**Objective:**

Whether short-term, low-potency opioid prescriptions for acute pain lead to future at-risk opioid use remains controversial and inadequately characterized. Our objective was to measure the association between emergency department (ED) opioid analgesic exposure after a physical, trauma-related event and subsequent opioid use. We hypothesized ED opioid analgesic exposure is associated with subsequent at-risk opioid use.

**Methods:**

Participants were enrolled in AURORA, a prospective cohort study of adult patients in 29 U.S., urban EDs receiving care for a traumatic event. Exclusion criteria were hospital admission, persons reporting any non-medical opioid use (e.g., opioids without prescription or taking more than prescribed for euphoria) in the 30 days before enrollment, and missing or incomplete data regarding opioid exposure or pain. We used multivariable logistic regression to assess the relationship between ED opioid exposure and at-risk opioid use, defined as any self-reported non-medical opioid use after initial ED encounter or prescription opioid use at 3-months.

**Results:**

Of 1441 subjects completing 3-month follow-up, 872 participants were included for analysis. At-risk opioid use occurred within 3 months in 33/620 (5.3%, CI: 3.7,7.4) participants without ED opioid analgesic exposure; 4/16 (25.0%, CI: 8.3, 52.6) with ED opioid prescription only; 17/146 (11.6%, CI: 7.1, 18.3) with ED opioid administration only; 12/90 (13.3%, CI: 7.4, 22.5) with both. Controlling for clinical factors, adjusted odds ratios (aORs) for at-risk opioid use after ED opioid exposure were: ED prescription only: 4.9 (95% CI 1.4, 17.4); ED administration for analgesia only: 2.0 (CI 1.0, 3.8); both: 2.8 (CI 1.2, 6.5).

**Conclusions:**

ED opioids were associated with subsequent at-risk opioid use within three months in a geographically diverse cohort of adult trauma patients. This supports need for prospective studies focused on the long-term consequences of ED opioid analgesic exposure to estimate individual risk and guide therapeutic decision-making.

## Introduction

The opioid crisis continues despite substantial efforts to date [1, 2]. Opioid use disorder (OUD), with consequences including overdose, injection drug use, and impaired consciousness, is a massive contributor to morbidity, mortality, and economic burden [3–6]. Thus far, response to the opioid epidemic has predominantly focused on law enforcement and secondary/tertiary prevention, such as expanded OUD treatment [7–11] and overdose reversal [12], with reduced attention to primary prevention beyond opioid prescribing reductions [13–17].

Emergency departments (EDs) commonly encounter patients in pain [18–20], and EDs are a recognized source of opioid exposure [21, 22]. An initial opioid exposure is a necessary, if not sufficient, antecedent to OUD [23]. Moreover, it is accepted that widespread increases in opioid prescription lead to observed increases in opioid overdose [24–26]. Yet, whether this association is causal and the relative contribution of prescription opioid use to later OUD are poorly understood [21, 22, 27], particularly for short-term, low dose exposures in episodic, unscheduled care settings treating acute pain, such as the ED [16, 28, 29]. Further complicating this narrative, self-reported sources of early opioid exposure by individuals with OUD are subject to recall bias and case-control study designs cannot be used to estimate exposure risk for individuals not yet suffering from OUD [21, 30, 31]. Retrospective reports associating duration and dosage of initial opioid therapy with later long-term use [27, 32] do not assess non-medical use or otherwise distinguish at the time of follow-up whether opioids are for new painful conditions, chronic pain, or OUD.

Our objective was to use existing data from a multi-center, prospective, observational study of posttraumatic neuropsychiatric sequelae to evaluate the degree to which an analgesic opioid exposure in the ED contributes to at-risk opioid use. We hypothesized that opioid exposure during the initial ED encounter for a traumatic event would be associated with at-risk opioid use within three months.

## Materials and methods

### Study design and setting

This study was a secondary analysis of data collected during the AURORA (Advancing Understanding of RecOvery afteR traumA) study. AURORA collected a wide array of psychological and biobehavioral data from adult patients recruited from a geographically diverse sample of 29 urban, U.S. emergency departments (EDs) who presented within 72 hours of a physical trauma [33]. Detailed elsewhere [33], participants provided written informed consent and completed baseline surveys in the ED and completed follow-up surveys at 2-weeks, 8-weeks, and 3-months after the initial visit. AURORA participants were: a) 18–65 years old, b) able to speak and read English, c) without cognitive impairment, d) able to use their own smart phone for >1 year post-enrollment, and e) discharged home or hospitalized for fewer than three days. Patients were excluded for solid organ injury > Grade 1 as defined by the American Association for the Surgery of Trauma (AAST), significant hemorrhage, requiring a chest tube or operation with anesthesia, or receiving greater than 20 morphine milligram of opioid medication daily prior to enrollment [34]. Occupational, self-inflicted, and injuries related to domestic violence were also excluded. The study was centrally approved by the Institutional Review Board at UNC Chapel Hill (IRB#17–0703), and all participants provided written informed consent.

### Participant selection

This analysis included AURORA participants enrolled after September 2017 who completed the 3-month follow-up assessment by October 2019. We additionally excluded from analysis those reporting any non-medical opioid use in the 30 days before enrollment and those with missing or incomplete opioid use/exposure responses or pain scores.

### Main outcomes/measures

We developed a composite definition using surrogate markers of interest to identify a group of patients spanning from high to potential concern. The primary outcome was “at-risk opioid use” defined as the composite outcome of 1) any non-medical opioid use after the initial ED visit (at 2-week, 8-week, or 3-month follow-up), or 2) opioid prescription use at 3-month follow-up. Non-medical opioid use was defined by affirmative response to the survey question “heroin, any opioids without a prescription, or taking more than prescribed for euphoria” [35, 36] This definition of “at-risk” use depends on a simplifying assumption that 1) any non-medical opioid use is problematic, and 2) pain at three months would generally be due to the original traumatic event with a transition to chronic pain and that 3) ongoing opioid exposure for chronic pain (3 months or greater) is at-risk for disordered opioid use [35, 37, 38]. Most traumatic injuries have healed to the degree possible absent additional complications by three months, and long-term prescription opioid use is associated with negative outcomes [3, 4, 6, 38, 39].

Exposure was measured as opioid administration for analgesia only during the ED visit, a prescription for opioids at ED discharge, or both at study enrollment. Covariates included self-reported patient gender, sex at birth, age, race/ethnicity, pain score at baseline and at 3-month follow-up, prescription opioid use in the 30 days prior to enrollment, marital status, employment status, income, injury severity score at baseline, and self-reported history of opioid use disorder.

### Primary data analysis

Descriptive statistics were used to summarize and assess participant selection characteristics. Summary data are reported as percentages, percentages with 95% confidence intervals (CI), medians with interquartile range (IQR), and means with 95% CI. Crude (unadjusted) odds ratios (cORs) and adjusted ORs (aORs) are presented with 95% CI to assess statistical significance.

We first conducted univariable analyses to quantify the association between at-risk opioid use and ED opioid exposure (none, ED prescription only, ED administration for analgesia only, ED administration and prescription), as well as a wide array of potential confounders of this association and possible risk factors for at-risk opioid use leveraging literature and expert opinion [29, 40, 41]. We then used multivariable logistic regression to further characterize the relationship between ED opioid exposure and at-risk opioid use, accounting for potential confounders and risk factors. All variables from the univariable analysis with a (p≤0.10) association via Fisher’s exact test for categorical data, Student’s t-test for parametric data, or Kruskal-Wallis test for non-parametric continuous data were included in an initial multivariable model. ED opioid exposure was kept in all models regardless of p-value. We used backward elimination to remove covariates with a p-value >0.05 starting with the covariate with the highest p-value based on a likelihood ratio test. All excluded covariates were re-introduced one at a time to assess confounding between ED opioid exposure and at-risk opioid use. Variables that resulted in a change in the regression coefficient of ≥10% were considered significant and included in the final model. After identifying the preliminary final model, goodness-of-fit, discrimination, and diagnostic statistics were calculated. The assumption of a linear relationship between the outcome and continuous variables in the logit (log-odds) scale was tested using fractional polynomials and graphic analyses. We also examined for clinically plausible interactions (i.e., effect modification) between ED opioid exposure and previous prescription that might affect the relationship between ED opioid exposure and at-risk opioid use; however, we found no evidence of significant effect modification. We conducted sensitivity analyses to assess the robustness of primary analysis results. Potential outliers and overly influential observations identified via diagnostic statistics were checked for miscoding and removed as part of these sensitivity analyses.

## Results

### Study flow and participant characteristics

**Fig 1:**
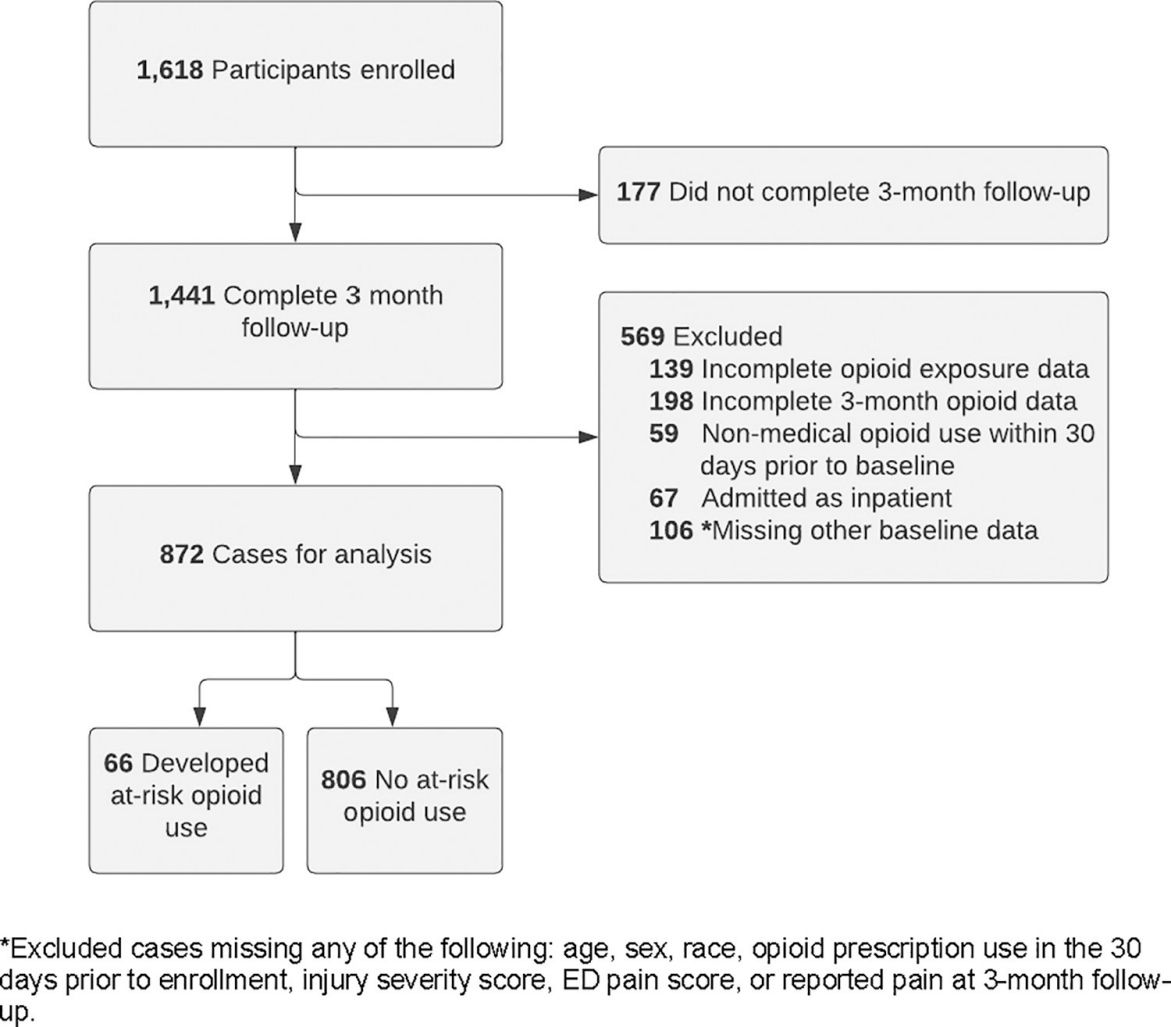
Enrollment, follow-up, and exclusion criteria flow diagram for persons in analysis.

There were 1441 patients available for analysis in the AURORA 3-month follow-up cohort, and subsequent exclusion criteria are outlined in Fig 1. We excluded 569 participants for the following reasons: 1) missing the primary outcome at two weeks, eight weeks, or three months (n = 198), 2) missing ED opioid exposure data during enrollment visit (n = 139), 3) missing age, sex, race, history of opioid prescription use in the 30 days prior to enrollment, injury severity score, ED pain score, or reported pain at 3-month follow-up (n = 106), 4) reported non-medical opioid use prior to enrollment (n = 59), and 5) hospitalized at conclusion of ED encounter (n = 67). Participants’ demographic characteristics, medical history, and ED opioid exposure are stratified by at-risk opioid use versus no at-risk use in Table 1. Of the 872 participants with complete data in the analysis, 54% were Black/African American, 67% female, and median age was 34 years.

**Table 1:** Characteristics of study population.

	**Total Included Participants**		**At-Risk Opioid Use**		**No At-Risk Opioid Use**	
	**N = 872**	**(%)**	**N = 66**	**(%)**	**N = 806**	**(%)**
**Baseline Characteristics**						
**Age–years, median (IQR)**	34	(26–46)	42	(33–48)	33	(25–46)
**Race/Ethnicity**						
White, Non-Hispanic	101	(11.6)	12	(18.2)	89	(11.0)
Hispanic	267	(30.6)	15	(22.7)	252	(31.3)
Black/African American	471	(54.0)	36	(54.5)	435	(54.0)
Other	33	(3.8)	3	(4.5)	30	(3.7)
**Sex–**Male	285	(32.7)	20	(30.3)	265	(32.9)
**Body Mass Index, median (IQR)**	29.3	(24–35)	30.6	(23.7–36.1)	29.1	(24–35)
**Opioid RX in 30 days prior to enrollment**	25	(2.9)	8	(12.1)	17	(2.1)
**Maximum Pain Severity Prior 30 Days**						
Moderate/Severe (4–10)	579	(66.6)	33	(50.0)	546	(67.9)
**Lifetime History of OUD**	74	(8.5)	14	(21.2)	60	(7.4)
**Lifetime History of Alcohol Use Disorder**	579	(66.4)	38	(57.6)	541	(67.1)
**Marital Status**						
Married	194	(22.2)	16	(24.2)	178	(22.1)
Divorced or Separated	145	(16.6)	19	(28.8)	126	(15.6)
Widowed	13	(1.5)	0	(0.0)	13	(1.6)
Never Married/Not reported	520	(59.6)	31	(47.0)	489	(60.7)
**Education**						
No Highschool Diploma	111	(12.7)	14	(21.2)	97	(12.0)
Highschool Diploma, GED/Equivalent	211	(24.2)	20	(30.3)	191	(23.7)
Some College/Associate Degree	354	(40.6)	22	(33.3)	332	(41.2)
Bachelor’s Degree	129	(14.8)	6	(9.1)	123	(15.3)
Graduate/Professional degree	67	(7.7)	4	(6.0)	63	(7.8)
**Employment Status at Week 2 Follow-up**						
Employed	644	(73.9)	39	(59.1)	605	(75.1)
Retired/Homemaker	37	(4.2)	7	(10.6)	30	(3.7)
Student	34	(3.9)	0	(0.0)	34	(4.2)
Unemployed, disabled, other	157	(18.0)	20	(30.3)	137	(17.0)
**Family Income at Week 2 Follow-up**						
Less than or equal to $19,000	303	(34.7)	29	(43.9)	274	(34.0)
$19,001 - $35,000	262	(30.0)	19	(28.8)	243	(30.1)
$35,001 - $50,000	125	(14.3)	6	(9.1)	119	(14.8)
$50,001 - $75,000	63	(7.2)	5	(7.6)	58	(7.2)
$75,001 or greater	105	(12.0)	4	(6.1)	101	(12.5)
Not reported	14	(1.6)	3	(4.5)	11	(1.4)
	**Total Included Participants**		**At-Risk Opioid Use**		**No At-Risk Opioid Use**	
	**N = 872**	**(%)**	**N = 66**	**(%)**	**N = 806**	**(%)**
**Characteristics at Enrollment**						
**ED Opioid Exposure**						
None	620	(71.1)	33	(50.0)	587	(72.8)
Prescription only	16	(1.8)	4	(6.1)	12	(1.5)
Administration only	146	(16.7)	17	(25.8)	129	(16.0)
Prescription & Administration	90	(10.3)	12	(18.2)	78	(9.7)
**Pain at ED Visit—0–10, median (IQR)**	7	(5–9)	8	(7–9)	7	(5–8)
**Maximum Abbreviated Injury Score (1–6)**						
1	745	(85.5)	60	(90.9)	685	(85.0)
2	123	(14.1)	6	(9.1)	117	(14.5)
3+	4	(0.5)	0	(0)	4	(0.5)
**Traumatic Event Types**						
Motor Vehicle Collision	822	(94.3)	66	(100.0)	756	(93.8)
Physical Assault	17	(1.9)	0	(0)	17	(2.1)
Fall >10 Feet	33	(3.8)	0	(0)	33	(4.1)
**Presence of Spinal Injury**						
No Spinal Injury	459	(52.6)	36	(54.6)	423	(52.5)
Injury, No Fracture	393	(45.1)	27	(40.9)	366	(45.4)
Fracture	20	(2.3)	3	(4.6)	17	(2.1)
	**All Complete Cases**		**At-Risk Opioid Use**		**No At-Risk Opioid Use**	
	**N = 872**	**(%)**	**N = 66**	**(%)**	**N = 806**	**(%)**
**Outcomes Characteristics at 3 months**						
**Opioid Use**						
No At-Risk Opioid Use	806	(92.4)	0	(0)	806	(100)
Non-Medical Use Only	44	(5.0)	44	(66.7)	0	(0)
Prescription Use Only	16	(1.8)	16	(24.2)	0	(0)
Both Prescription and Non-Medical Use	6	(0.7)	6	(9.1)	0	(0)
**Pain Severity**						
Moderate/Severe (4–10)	375	(43.0)	13	(19.7)	362	(44.9)
No Pain/Minor (0–3)	497	(57.0)	53	(80.3)	444	(55.1)

Abbreviations: IQR, interquartile range; ED, emergency department; RX, prescription.

*At-Risk opioid use defined as *either* self-reported 1.) non-medical opioid use at 2-week, 8-week, or 3-month follow-up or 2.) prescription opioid use at 3- month follow-up.

### Primary outcome

Of 872 subjects included in the primary analysis, at-risk opioid use was reported by 66 (7.6%) individuals by the 3-month follow-up. In comparison to type of ED opioid exposure, at-risk opioid use was reported by: 33/620 (5.3%, CI: 3.7, 7.5) without an ED opioid analgesic exposure, 4/16 (25%, CI: 8.3, 52.6) with ED opioid prescription only, 17/146 (11.6%, CI: 7.1, 18.3) with in-ED opioid administration only, and 12/90 (13.3%, CI: 7.4, 22.5) with both ED administration and opioid prescription at discharge (Table 1).

### Multi-variable analysis

Compared to no ED opioid exposure, the aOR for at-risk opioid use was 4.9 (CI 1.4, 17.4) for ED opioid prescription only, 2.0 (CI 1.0, 3.8) for ED administered opioids only, and 2.8 (CI 1.2, 6.5) for both ED opioid administration and prescription at discharge, when controlling for patient age, prescription opioid use prior to enrollment, pain at initial ED visit, moderate or severe pain at three months, race/ethnicity, marital status, injury severity score, and self-reported history of OUD (Table 2). Other patient characteristics (e.g., income, education, employment status, gender) were not associated with at-risk opioid use in the multivariable model (Table 2). The aOR for at-risk opioid use did not differ significantly for ED opioid prescriptions only compared to either ED-administration only (aOR 2.0, CI 0.5, 7.2) or ED-administration plus prescription at discharge (aOR 1.7, CI 0.4, 6.5). Combining all ED opioid exposure categories suggested exposure to any opioid (prescription at discharge or in-ED administration, or both) during the ED visit was associated with a more than doubling of the odds of at-risk opioid use by the 3-month follow-up period (aOR 2.2, CI 1.3, 3.75). Sensitivity analyses were consistent with primary findings.

**Table 2:** Logistic regression model for at-risk opioid use during 3-month follow-up after ed visit for trauma.

Risk Factor	At-Risk Opioid Use*			
	n/N	Percent (95% CI) / Median (IQR)	Unadjusted OR (95% CI)	Adjusted** OR (95% CI)
**ED Opioid Exposure**				
No ED exposure	33/620	5.3 (3.8, 7.3)	REFERENT	REFERENT
ED Administration only	17/146	11.6 (7.2, 17.6)	2.42 (1.36, 4.30)	1.96 (1.01, 3.81)
Prescription & ED Administration	12/90	13.3 (7.5, 21.5)	2.99 (1.62, 5.55)	2.79 (1.20, 6.49)
Prescription only	4/16	25.0 (9.1, 49.1)	5.93 (1.81, 16.4)	4.90 (1.38, 17.38)
**Previous Opioid Rx within 30 days**				
No	58/847	6.8 (5.3, 8.7)	REFERENT	REFERENT
Yes	8/25	32.0 (16.4, 51.5)	6.40 (2.65, 15.46)	3.11 (1.13, 8.57)
**Pain Score at Enrollment (0–10), per 1-point increase**	N = 872	7 (5–9)	1.26 (1.12, 1.42)	1.18 (1.04, 1.35)
**Pain Score at 3-Months (0–10 scale)**				
No Pain/Minor Pain (0–3)	13/375	3.5 (2.0, 5.7)	REFERENT	REFERENT
Moderate/Severe Pain (4–10)	53/497	10.7 (8.2, 13.6)	3.32 (1.78, 6.19)	3.00 (1.54, 5.84)
**Age at Enrollment, per 5-year increase**	N = 872	34 (26–47)	1.14 (1.04, 1.25)	1.13 (1.03, 1.26)
**Race/Ethnicity**				
White, non-Hispanic	15/267	5.6 (3.3, 8.9)	REFERENT	REFERENT
Hispanic	12/101	11.9 (6.7, 19.2)	2.27 (1.02, 5.02)	2.65 (1.11, 6.31)
Other	39/504	7.7 (5.6, 10.3)	1.41 (0.76, 2.61)	1.23 (0.61, 2.45)
**Lifetime History of Opioid Use Disorder**				
No	52/798	6.5 (5.0, 8.4)	REFERENT	REFERENT
Yes	14/74	18.9 (11.3, 28.9)	3.35 (1.75, 6.39)	4.39 (2.14, 9.03)
**Maximum Abbreviated Injury Score**				
2 or greater	6/127	4.7 (2.0, 9.5)	REFERENT	REFERENT
1	60/745	8.1 (6.3, 10.2)	1.77 (0.75, 4.18)	2.80 (1.05, 7.48)

*At-Risk opioid use defined as prescription opioid use at 3 months or any non-medical opioid use after ED visit.

**Adjusted for all variables in table; Hosmer-Lemeshow GOF p-value = 0.84; calibration belt p-value = 0.49; area under the receiver operating characteristics curve = 0.793 (95% CI: 0.740, 0.845)

**Abbreviations**: CI, confidence interval; ED, emergency department; IQR, interquartile range; OR, odds ratio; Rx, prescription

## Discussion

Exposure to opioids after a traumatic event was associated with increased at-risk opioid use within three months in a geographically diverse cohort of patients who experienced trauma. While the study was observational, data were collected prospectively, and the associations of prescription opioid exposure and at-risk opioid use persisted after controlling for patient and clinical factors. Not surprisingly, ED opioid administration and prescribing was relatively common for these trauma patients, with 12% receiving an opioid prescription at ED discharge, and 29% receiving a prescription, in-ED administration, or both. These percentages equate to millions of exposures annually nationwide. Every year, approximately 35 million ED visits result from injury in the US, so even a small effect from prescription opioid exposure would have significant ramifications for individual and public health [42]. If even a small proportion of those exposures are causally related and avoidable, there is an urgent need to develop, target, and deploy efficacious interventions.

Our findings align with previous studies associating the strength and duration of initial opioid prescription with later use [21, 27, 32, 43, 44]. This study is unique in at least four respects. First, we considered both in ED administration and prescription at discharge. The hypothesis that administration in the ED without ongoing prescription exposure could influence long-term opioid-related outcomes contributes to our understanding of the development of OUD. Second, we assessed for and excluded individuals with self-reported previous non-medical opioid use from the cohort. As such, the cohort selected helps isolate new from ongoing at-risk opioid use. Third, we were able to control for patient and clinical factors that affect the association between exposure and outcome. Finally, we measured non-medical use as part of the outcome, as opposed to only continued usage. We do acknowledge that inclusion of opioid prescriptions at three months suffers from the same limitations as other retrospective longitudinal studies of prescription history. However, our use of a 3-month time horizon increases the likelihood that opioid use at three months was a continuation from the index event rather than a new and unrelated (and thus less concerning) short-term exposure.

When controlling for the effects of prior opioid exposure, opioid prescription in the 30 days prior to enrollment was associated with later at-risk opioid use (aOR 3.11, CI 1.13, 8.57). We do not know if this association was due to misclassification (i.e., undisclosed/undiagnosed opioid use disorder), a direct cause of increased risk, or a marker of pain or opioid response predisposing to at-risk use. It is easy to hypothesize that treatment for acute pain can be a causal event along the trajectory from initial exposure to later at-risk use even if not a triggering event when occurring as a first exposure.

Even if short-term low-potency opioid exposure is causally associated with later long-term opioid use, or the development of OUD, it does not mean that initial opioid exposure is necessarily avoidable. All therapy in medicine is associated with potential risks and benefits, and the potential for later harm must be balanced against the potential for unrelieved short-term suffering. Prospective study of opioid exposure for acute pain is necessary so that patients and providers can accurately estimate individualized risk to guide therapeutic decision-making. It is important to realize that scientific developments in this area could simultaneously support both expansions and reductions in opioid therapy depending on the individual patient and the circumstance.

### Limitations

While this study capitalized on the availability of a prospective, multicenter cohort, results should be understood in context with important limitations. Most notably, the generalizability of this analysis was limited to measures and sample size available from the parent study which was not specifically designed to assess opioid exposure or long-term opioid use. Bias may have been introduced by our exclusion of a large number of participants with missing follow-up data and well as the voluntary aspect of research participation. These exclusions may limit the number of individuals with later at-risk opioid use due to stigma and the self-reported nature of the survey follow-up. Additionally, due to sample size limitations, we used a composite outcome that did not fully resolve limitations of prior studies in which the reasons for later prescription opioid use are uncharacterized. Our ability to assess causality is additionally limited by its observational design. Finally, due to the categorical nature of of both prior opioid prescription use and later opioid use, we were unable to reduce time-based confounding in our model.

## Conclusions

Exposure to opioids from an ED visit was associated with increased odds of at-risk opioid use within three months among trauma patients when controlling for age, gender, race/ethnicity, prescription opioid use prior to enrollment, pain, injury severity score, and self-reported history of OUD. These results support the need for prospective study focused on the long-term consequences of ED opioid analgesic exposure to guide therapeutic decision-making.
